# Intravenous Administration of Allogenic Cell-Derived Microvesicles of Healthy Origins Defends Against Atherosclerotic Cardiovascular Disease Development by a Direct Action on Endothelial Progenitor Cells

**DOI:** 10.3390/cells9020423

**Published:** 2020-02-12

**Authors:** Nicoleta Alexandru, Eugen Andrei, Florentina Safciuc, Emanuel Dragan, Ana Maria Balahura, Elisabeta Badila, Adriana Georgescu

**Affiliations:** 1 Institute of Cellular Biology and Pathology ‘Nicolae Simionescu’ of Romanian Academy, 030167 Bucharest, Romania; nicoleta.alexandru@icbp.ro (N.A.); eugen.andrei.dev@gmail.com (E.A.); florentina.safciuc@icbp.ro (F.S.); emanuel.dragan@icbp.ro (E.D.); 2 ‘Carol Davila’ University of Medicine and Pharmacy, 050474 Bucharest, Romania; daraban.m.a@gmail.com (A.M.B.); elisabeta.badila@gmail.com (E.B.); 3 Internal Medicine Clinic, Emergency Clinical Hospital, 014461 Bucharest, Romania

**Keywords:** cell-derived microvesicles (MVs), endothelial progenitor cells (EPCs), microRNAs, atherosclerosis, therapy

## Abstract

Atherosclerosis and cardiovascular disease development is the outcome of intermediate processes where endothelial dysfunction and vascular inflammation are main protagonists. Cell-derived microvesicles (MVs), endothelial progenitor cells (EPCs), and circulating microRNAs (miRNAs) are known as biomarkers and potential regulators for atherosclerotic vascular disease, but their role in the complexity of the inflammatory process and in the mechanism of vascular restoration is far from clear. We aimed to evaluate the biological activity and functional role of MVs, in particular of the EPCs-derived MVs (MVEs), of healthy origins in reducing atherosclerotic vascular disease development. The experiments were performed on hamsters divided into the following groups: simultaneously hypertensive–hyperlipidemic (HH group) by combining two feeding conditions for 4 months; HH with retro-orbital sinus injection containing 1 × 10^5^ MVs or MVEs from control hamsters, one dose per month for 4 months of HH diet, to prevent atherosclerosis (HH-MVs or HH-MVEs group); and controls (C group), age-matched normal healthy animals. We found that circulating MV and MVE transplantation of healthy origins significantly reduces atherosclerosis development via (1) the mitigation of dyslipidemia, hypertension, and circulating EPC/cytokine/chemokine levels and (2) the structural and functional remodeling of arterial and left ventricular walls. We also demonstrated that (1) circulating MVs contain miRNAs; this was demonstrated by validating MVs and MVEs as transporters of Ago2-miRNA, Stau1-miRNA, and Stau2-miRNA complexes and (2) MV and MVE administration significantly protect against atherosclerotic cardiovascular disease via transfer of miR-223, miR-21, miR-126, and miR-146a to circulating late EPCs. It should be mentioned that the favorable effects of MVEs were greater than those of MVs. Our findings suggest that allogenic MV and MVE administration of healthy origins could counteract HH diet-induced detrimental effects by biologically active miR-10a, miR-21, miR-126, and miR-146a transfer to circulating EPCs, mediating their vascular repair function in atherosclerosis processes.

## 1. Introduction

Atherosclerosis is one of the most important causes of death in the world, and at the same time it is a trigger for cardiovascular disease. Atherosclerosis, also known as systemic disease, has significant consequences on important organs and tissues such as the brain, kidneys, mesentery, and limbs. Atherosclerosis, as a chronic inflammatory disease of the arterial wall, occurs as a result of subendothelial lipoprotein retention, endothelial activation, and migration of immune cells to inflamed intima that then generate the formation of fatty streaks and subsequent atheromas [1,2]. It has been repeatedly shown that cell-derived microparticles (MPs) or microvesicles (MVs) play an extremely important role in the initiation, progression, and development of clinical complications of cardiovascular disease; they appear to be an important biomarker in predicting this pathology [3,4,5]. Also, a previous study showed that the ratio of circulating MVs to endothelial progenitor cells (EPCs) could be considered a biomarker of vascular dysfunction and a precursor of atherosclerosis [6]. Most studies have described MPs or MVs as small (0.1–1.5 µm), pro-inflammatory vesicles released by many cell types (e.g., endothelial cells, platelets, monocytes, leucocytes) in a closely controlled process. MPs and MVs have also been shown to contain cytoplasm and surface markers of their origin cells [7,8,9]. With regard to their life after release into circulation by the cells, it is assumed that they bind and fuse with target cells through receptor-ligand interactions, and then, through their cellular content, MVs modulate a series of biological processes, mediating in this way the complex processes of vascular inflammation and coagulation [10]. Increasing evidence indicates that inflammatory and procoagulatory effects of MVs on their target cells are due to their rich lipid composition and other proinflammatory components originating from the cells of origin [7]. It was also shown that MVs enclose messenger RNAs (mRNAs) and microRNA (miRNAs) that they carry to the target cells, modulating their protein expression [11,12]. An important question is whether MVs contain other miRNAs than those originating from their home cells. Since more than 1000 different human miRNAs have been discovered, the interaction between miRNAs and mRNAs is highly complex and currently not completely understood. However, approximately one-third of human protein-encoding genes are miRNAs-regulated, underlining the extraordinary impact of miRNAs on protein expression [13,14]. Recent data indicate that miRNAs can be found in circulating blood but also in cardiac tissue, and for this reason they are considered to have a defining role in cardiovascular diseases [15,16,17,18,19]. Interestingly, alterations in the expression of specific miRNAs in human atherosclerotic plaques have been reported [20,21,22,23]. These suggest that miRNAs may have a vital involvement in atherosclerotic plaque advancement toward instability and rupture. Furthermore, it was suggested that MPs or MVs represent transport vehicles for many of the miRNAs in circulation [24,25]. Taken together, these observations open new research perspectives in the circulating MV and miRNA fields.

Therefore, this study aims to determine which are the biochemical, structural, and functional changes relevant for our atherosclerotic model and to evaluate the possible protective role of MVs, in particular of the EPCs-derived MVs (MVEs), of healthy origins in atherosclerosis development as well as the mechanisms responsible for their repair capacity.

Our results will support the idea that MVs, particularly EPC-derived MVs (MVEs), have the ability to carry a specific class of miRNAs in circulation and transfer them to EPCs and the endothelial cell (EC) layer through a tightly regulated process. In addition, we will show that MVs and MVEs are able to program stem/progenitor cells to repair tissue injury after miRNA transfer, suggesting that they could be exploited as a possible therapeutic method for regenerative medicine in cardiovascular disease.

## 2. Materials and Methods

### 2.1. Generation of the Experimental Model of Atherosclerosis, Including Transplant of MVs and MVEs

#### 2.1.1. Achievement of the Experimental Animal Models

**Figure 1:**
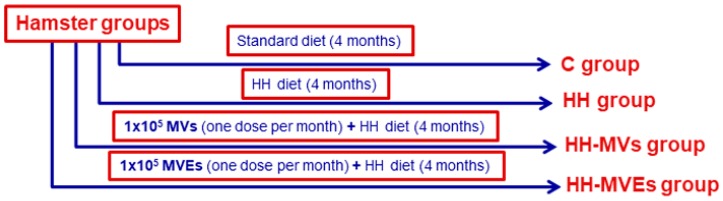
Graphic representation of animal experimental models. Golden Syrian hamsters were divided into four experimental groups: (**1**) control (C) group; (**2**) simultaneously hypertensive–hyperlipidemic (HH) group; (**3,4**) HH hamsters with retro-orbital sinus injection containing **1 × 10^5^** microvesicles (MVs) (HH-MVs group) or **1 × 10^5^** endothelial progenitor cell (EPC)-derived MVs (MVEs) (HH-MVEs-group).

The Golden Syrian hamsters (120 male, 3 months old, 115.2 ± 2.5 g body weight) were organized in four groups: (**1**) simultaneously hypertensive-hyperlipidemic (**HH group**) obtained after a diet enriched in 3% cholesterol, 15% butter, and 8% NaCl, and administered for 4 months to induce atherosclerosis; (**2,3**) HH with retro-orbital sinus injection containing **1 × 10^5^ MVs or EPC-derived MVs** (**MVEs**) from control hamsters, and given in a single dose per month for 4 months of the HH diet to prevent atherosclerosis (**HH-MVs or HH-MVEs groups**); and (**4**) **controls** (**C group**), healthy animals of the same age grown in the same conditions and receiving a standard diet containing basal 1% NaCl (Figure 1).

Depending on the type of experiment, animals were anesthetized with 2% isoflurane (for blood collection and the Vevo 2100 imaging system) or a combination of ketamine/xylazine/acepromazine (80 mg/10 mg/2 mg/kg body weight) for tissue collection.

All the experimental procedures were carried out in the Institute of Cellular Biology and Pathology ‘Nicolae Simionescu’. They were approved by the Ethics Committees of the Institute according to Decision no. 1/15.03.2016, and were conducted in accordance with national, European, and international legislation on the use of experimental animals in biomedical research.

#### 2.1.2. Isolation and Purification of the Total Circulating MVs from Control Plasma

The peripheral blood from the C group was collected on 0.138 M tri-sodium citrate 9/1 (*vol/vol*) with a spin-down at 2500 g for 10min at 4 °C to collect the platelet-poor plasma (PPP) from the supernatant. Subsequently, PPP was centrifuged at 13,000× *g* for 2 min at 4 °C to remove residual platelets, apoptotic bodies, and to collect the platelet-free plasma (PFP) in the supernatant. Finally, MVs were isolated by PFP centrifugation at 20,000× *g* for 90 min at 4 °C. Pelleted MVs were washed twice (20,000× *g*, 90 min, 4 °C, to minimize the effects of any remnant-soluble mediators) and re-suspended in phosphate buffered saline (PBS) [26].

#### 2.1.3. Characterization, Quantification, and Sorting of the MVs and MVEs

The MVs purified above and in suspension (20 µL) were labeled with AnnexinV-FITC (which bind phosphatidylserine (PS)) to sort total MVs, or with Annexin V-FITC and anti-CD34-PE (which bind CD34 on EPCs) to sort MVEs. The quantification and sorting were performed after 40 min incubation time, at room temperature (RT) in the dark [4,6]. Fluorescent beads with diameters of 0.5, 0.9, and 3 µm were used for calibration and antibodies IgG2a-FITC and IgG1-PE were used as negative control for setting up the machine voltages. For these experiments, a flow cytometer MoFlo (Dako, Carpinteria, CA, USA) equipped with a high-speed cell sorter was used. For cell sorting by flow cytometry, the speed was about 10,000–12,000 MVs/sec.

#### 2.1.4. Verification of the Injection with MVs and MVEs

To trace in vivo MVs or MVEs, these were stained with PKH26 (2 × 10^−6^ M) [10] and administrated to HH hamsters in a single injection for 3 days. The local infiltration of MVs or MVEs to different target organs (liver, kidney, lung, brain, heart, thoracic aorta, and mesenteric resistance arteries) was quantified under IVIS spectrum equipment. For PKH26, the excitation wavelength was 535 nm and emission wavelengths were 580, 600, 620, 640, and 660 nm; vesicles (MVs or MVEs) stained with these membrane-fluorescent dyes were observed by using the IVIS at the same wavelengths. The analysis of obtained images was performed by using Living Image 4.4. Software (Caliper Life Sciences, Waltham, MA, USA). An automated spectral distribution algorithm (called Spectral unmixing) was used. This kind of analysis should separate the specific signal originating from a particular fluorochrome and the background signal originating from the autofluorescence and food. The fluorescence-specific signal was shown as the radiant efficiency, Emission light (photons/sec/cm2/str)/Excitation light (μW/cm^2^), resulting from the manual setting ranges of minimum and maximum fluorescence strength. The ranges were selected on the basis of control images (PBS-injected animal) and images obtained for an animal injected with PKH26-labeled vesicles.

### 2.2. Characterization of the Experimental Animal Models

#### 2.2.1. Analysis of the Plasma Parameters

After 4 months of the hyperlipemic–hypertensive diet and treatment, the plasma glucose, cholesterol, and triglyceride concentrations were assayed using the Accutrend GCT apparatus. The systolic and diastolic arterial blood pressure and the heart rate were measured with a blood pressure device and an advanced physiological monitoring unit, both connected to Vevo2100 imaging system (VisualSonics).

#### 2.2.2. Blood Pressure and Heart Rate Measurements

To diagnose hypertension for our HH model, systolic and diastolic blood pressures and also heart rate were measured under a Physiological Pressure Transducer (model MLT844/D) connected to a PowerLab data acquisition unit.

#### 2.2.3. Exploration of the Structural, Architectural, and Flow Changes

To see arterial wall disorders induced by the hyperlipemic–hypertensive diet and treatment, distensibility, wall thickness, and pulse wave velocity were measured for thoracic and carotid arteries by duplex ultrasonography in M, B, and PW Doppler Mode using Vevo2100. Subsequently, for characterization of the cardiac hypertrophy, the shortening fraction and relative wall thickness were measured for the left ventricle in M and B mode under Vevo2100. Before examination, each hamster was lightly anesthetized with 2% isoflurane.

#### 2.2.4. Isolation and Specific Quantification of the Circulating EPCs

(a) Preparation of the viable mononuclear cells (MNCs) from blood. Briefly, 1 mL whole blood was layered onto 3 mL Histopaque-1077 and centrifuged at 400× *g* for 30 min to be obtained the opaque interface containing MNCs. Subsequently, the opaque interface was transferred into a clean tube and 10 mL PBS were added, mixed, centrifuged at 250× *g* for 10 min, and the supernatant was aspirated and discarded. This washing was repeated three times and the obtained MNC pellet was resuspended in 10 mL PBS [27]. If the obtained MNC fraction was not well washed, the ammonium-chloride-potassium (ACK) lysing buffer was added and the washing was repeated.

(b) Quantification of the circulating EPCs. EPC levels (in percentage (%)) were evaluated after the incubation of 10^4^ MNCs/100 μL PBS with specific antibodies against CD34-PE, CD133-APC, and KDR-FITC in the dark for 40 min at RT and further analysis through flow cytometry.

Negative control type experiments using specific isotype and dead cell quantification experiments using propidium iodide staining (4 μg/mL) were also performed under the flow cytometer [28,29].

#### 2.2.5. Analysis of the Plasma Cytokine and Chemokine Profiles by Enzyme-Linked Immunosorbent Assay (ELISA)

After 4 months of the experimental diet, the venous blood taken from the retro-orbital plexus was collected on 0.138 M tri-sodium citrate 9/1 (*vol/vol*). Within 15 min, the blood was centrifuged at 1000× *g* for 20 min and plasma (upper portion) was immediately separated and frozen to −70 °C until examination [30]. Plasma concentrations of VEGF, MCP-1, IL-6, IL-1β, Il-8, and CD40L were assayed by the classical ELISA method.

### 2.3. Investigation of the Vascular Structure and Function

#### 2.3.1. Examination of the Ultrastructure of Thoracic Aortas, Carotid Arteries and Mesenteric Resistance Arteries for All Experimental Groups

The morphology of the arterial wall was explored by hematoxylin-eosin staining, and the quantification of lipid accumulation in the arterial wall was investigated by oil red staining using a fluorescent microscope [6].

#### 2.3.2. Evaluation of the Vascular Wall Function for All Experimental Groups

Animals were euthanized and all of the thoracic aortas, carotid arteries, and mesenteric vascular beds were excised. The isolated segments (from aortas and resistance arteries) were mounted in wire-myograph [31]. The vascular wall responses to vasoconstrictor agonists, NA (10^−8^–^10−4^ M), 5-HT (10^−8^–10^−4^ M), and K^+^ (24.4 mM, 42.46 mM, 64.1 mM, 83.93 mM, 123.7 mM) and vasodilators agonists, Ach (10^−8^–10^−4^ M) and SNP (10^−8^–10^−4^ M), were explored by engaging the myograph technique [6].

### 2.4. Detection of the MVs and MVEs on Slides

For this purpose, 5 × 10^5^ MVs or MVEs in suspension were labeled with lipophilic dye PKH26 (2 × 10^−6^ M) (dye with diluent) for 3 min. Next, they were incubated with fetal bovine serum for 1 min and washed with PBS. After centrifugation at 400× *g* for 10 min at 25 °C, the MVs or MVEs pellet was suspended in PBS, spotted on glass slides, and viewed under a fluorescent microscope (Nikon E800 Fluorescence Microscope, Nikon, El Segundo, CA, USA) [4].

### 2.5. Identification of the miRNA-Binding Proteins (Ago2, Stau1, Stau2) in MVs and MVEs to Investigate the Mechanism of miRNA Transport

The 5 × 10^5^ MVs or MVEs were isolated and sorted in 300 µL PBS from the peripheral blood collected from the C group as described above. The indirect immunofluorescence was performed on 5 µL from each sample spotted on poly-*L* lysine coating slides and left for 30 min at RT. The samples spotted on glass slides were fixed in 2% paraformaldehyde (PFA) for 15 min, washed 3 times with PBS, permeabilized with 0.5% TritonX-100 in PBS for 15 min, and incubated overnight with primary antibodies for Ago2 (argonaute 2, a protein involved in miRNA transport and processing) and Stau 1 and 2 (staufen1/2, proteins involved in the transport and stability of mRNAs). After washings 3 times in PBS, MVs or MVEs were incubated for 60 min with secondary antibodies labeled with FITC or PE, respectively. Subsequently, after the staining and mounting with Fluoroshield and DAPI, the samples were viewed with a fluorescent microscope (Nikon E800 Fluorescence Microscope, Nikon, El Segundo, CA, USA). The presence of fluorescence suggested that MVs and MVEs shuttle the ribonucleoproteins involved in miRNA traffic.

### 2.6. Examination of the miRNA Profile in Circulating MVs, MVEs, and EPCs

The 1 × 10^6^ circulating MVs or MVEs were isolated and sorted from blood collected from C and HH groups, and 1 × 10^6^ circulating EPCs were isolated and sorted from blood collected from C, HH, HH-MVs, and HH-MVEs groups as described above, and the expressions of miRNAs (miR-21, miR-126, miR-146a, miR-223) with detrimental roles in cardiovascular disease were measured by real-time quantitative-PCR (RT q-PCR). The levels of hsa-miR-21-5p (ID: 000397), hsa-miR-126-3p (ID:002228), hsa-miR-146a-5p (ID:000468), and hsa-miR-223-3p (ID: 002295) were evaluated in MVs, MVEs, and EPCs by the RT q-PCR method, and further analysis of the data was completed according to the protocol described by Alexandru et al., 2017 using ViiA7 a real-time PCR system and ViiA7 Software v1.2 (Applied Biosystems, Life Technologies, Carlsbad, CA, USA).

### 2.7. RNA Integrity Analysis

The most common method, agarose gel electrophoresis (1% denaturing agarose gel), was used to assess the integrity of total RNAs [32]. The presence of 28S and 18S rRNAs was examined. The majority of total RNAs in MVs or MVEs were concentrated below 2 kb, which represents mRNAs and miRNAs.

### 2.8. Assessment of the mir-223 Expression in Circulating MVs and MVEs, and in Late EPC Cultures

The 1 × 10^5^ MVs or MVEs isolated and sorted from the blood collected from C group were incubated with a miR-223-3p Hu-Cy5 SmartFlare™ miRNA detection probe for 16 h in darkness and then examined by flow cytometry for the measurement of miR-223 expression. The unincubated samples with a miR-223-3p Hu-Cy5 SmartFlare™ miRNA detection probe were used to set the gates corresponding to a correct analysis of MVs or MVEs by flow cytometry.

In separate experiments, the late EPCs were obtained and expanded in culture from the peripheral blood isolated from the four experimental animal groups: C, HH, HH-MVs, and HH-MVEs [33]. The obtained late EPC cultures, 3 × 10^4^ late EPCs-C, late EPCs-HH, late EPCs-HH-MVs, or late EPCs-HH-MVEs, were incubated with a miR-223-3p Hu-Cy5 SmartFlare™ miRNA detection probe for 16 h in darkness and observed by fluorescence microscopy to evaluate miR-223 expression.

The final step in successfully analyzing flow cytometry data was setting gates to separate positive from negative events.

### 2.9. Reagents

The standard chemicals, reagents, and the specific antibodies were purchased from Sigma Chemical Co. (St. Louis, MO, USA), Santa Cruz Biotechonolgy (European Support Office, Heidelberg, Germany), Merk & Co (Kenilworth, NJ, USA), Lonza (Basel, Switzerland), R&D Systems (Minneapolis, MN, USA), Qiagen (Hilden, Germany), and from Thermo Fisher Scientific (Waltham, MA, USA). All others reagents were of analytical grade.

For flow cytometry used the following antibodies: Annexin V(FL)-FITC (Santa Cruz, sc-4252 FITC, anti-human, source: E. coli, c = 0.05 µg/µL); PE anti-human CD34 antibody (Biolegend, cod 343606, host species: mouse, c = 0.005 µg/µL); CD133/2(293C3)-APC antibody (Millteny, Order no. 130-090-854, anti-human, clone: 293C3, c = 10 µL/10^6^ cells); human VEGFR2/KDR/Flk-1 Fluorescein-conjugated antibody (R&DSystem, FAB357F, source: monoclonal mouse IgG1 clone # 89106, c = 10 µL/10^6^ cells); Goat F(ab’)2 Anti-Human IgG H&L- (Phycoerythrin) pre-adsorbed antibody (abcam-ab7006, host species: goat, dilution: 1/100); goat anti-human IgG (H+L) cross-adsorbed secondary antibody, FITC (Invitrogen-31531, host species: goat, dilution: 1/100).

### 2.10. Statistical Analysis

Mean values of measured parameters and standard deviations were calculated; statistical significance of the differences between groups was measured using one-way ANOVA (for echocardiographic, flow cytometry, IVIS, and wire myograph measurements), two-way ANOVA (for plasma parameters), or Student’s *t*-test (for immunostaining measurements) in GraphPad Prism 7 software (San Diego, CA, USA). A value of *p* < 0.05 was considered statistically significant.

## 3. Results

### 3.1. In Vivo Infiltration of MVs and MVEs in Different Target Organs and Peripheral Blood

The verification of the effectiveness of MV or MVE injections was made by two different analysis methods: IVIS imaging system and flow cytometry.

**Figure 2:**
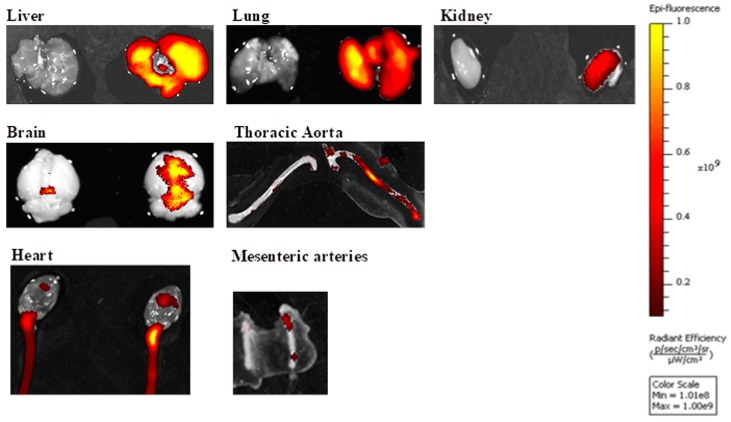
Representative images of organs/tissues (liver, lung, kidney, brain, heart, thoracic aorta, mesenteric resistance arteries) from the untransplanted HH group (on the left side of images in gray) and transplanted HH group (on the right side of images in red-yellow) analyzed with IVIS spectrum equipment for the presence of PKH26-labeled MVs or MVEs obtained by manual alignment of the spectrum. The color scale bar shows the range of strongest to weakest signal (1.01 × 10^8^–1.00 × 10^9^). The intensity is strongest for the yellow colored points. The darker the spot, the weaker the signal. The number of animals per experimental working group was equal to 4.

In separate experiments, to trace in vivo infiltration of MVs (1 × 10^5^) or MVEs (1 × 10^5^) into different organs and tissues, the plasma MVs/MVEs collected from C hamsters were stained with PKH26 (2 × 10^−6^ M) and administrated to HH hamsters in a single injection for 3 days in the first week of the HH diet. The infiltration of MVs or MVEs in some target organs such as livers, lungs, kidneys, brains, hearts, thoracic aortas, and mesenteric resistance arteries was followed and quantified under IVIS spectrum equipment for HH hamsters injected with MVs or MVEs (transplanted HH experimental group) compared to HH hamsters injected with PBS as a vehicle (untransplanted HH experimental group) (Figure 2 and Table 1). The aim of this procedure was to prove that MVs or MVEs were assimilated by different organs and tissues. It can be seen that radiant efficiency for PKH26 was significantly increased for all investigated organs/tissues from the transplanted HH experimental group (in red-yellow) compared to the untransplanted HH group (in gray), proving the effectiveness of injections of MVs or MVEs (Figure 2 and Table 1). Vehicle injection did not induce fluorescence in the analyzed wavelengths, which indicates the specificity of the signal detected in the transplanted HH group compared to the untransplanted HH group (Figure 2 and Table 1).

**Figure 3:**
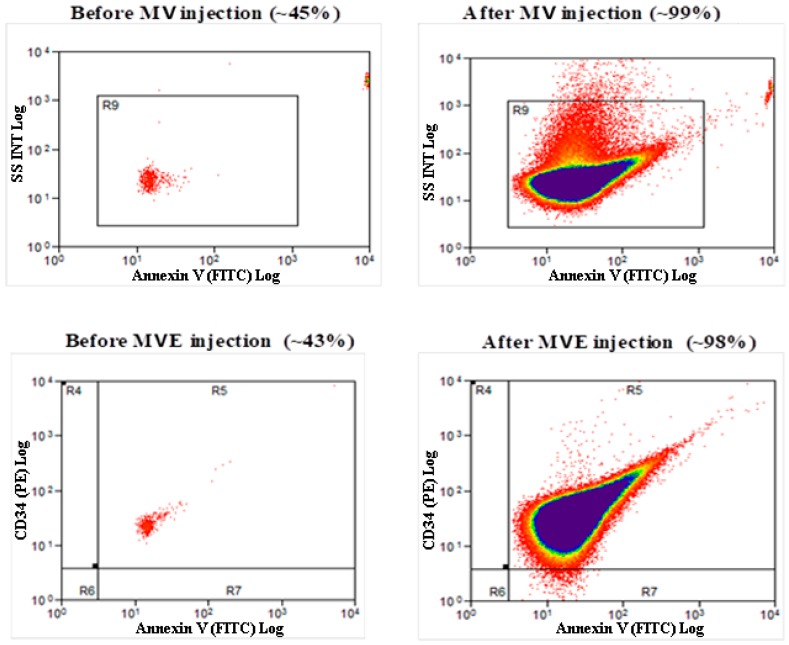
Representative flow cytometric measurements of MV/MVE levels before and after retro-orbital sinus injection containing 1 × 10^5^ MVs or MVEs to HH group (*n* = 3 individual animals per group); MVs were positive for AnnexinV-FITC, and MVEs were double positive for AnnexinV-FITC and CD34-PE.

In other experiments, MVs (1 × 10^5^) or MVEs (1 × 10^5^) from C hamsters were administrated by retro-orbital sinus injection to hamsters in the HH group in the first week of hypertensive–hypercholesterolemic diet. At 24 h after their transplantation, the levels of MVs positive for AnnexinV-FITC and MVEs double positive for AnnexinV-FITC and CD34-PE were evaluated in peripheral blood by flow cytometry. Analysis of flow cytometry data revealed significantly increased percentages for both plasma MVs (99% ± 7%) and MVEs (98% ± 9%) at 24 h after transplantation compared to the initial moment, before MV/MVE allogeneic transplantation, where the percent for MVs positive for AnnexinV-FITC was 45% ± 4%, and for MVEs double positive for AnnexinV-FITC and CD34-PE it was ~43% ± 4% (Figure 3). Again, these results showed the effectiveness of MV or MVE injections (Figure 3).

### 3.2. Efficacy of MV and MVE Administration on Blood Parameter Changes and Arterial and Left Ventricular Wall Disorders

At the beginning of the experimental period and immediately after randomization into groups, the blood glucose, cholesterol, and triglyceride levels were not different between the experimental groups: C, HH, HH-MVs, or HH-MVEs.

Importantly, the plasma glucose levels did not change over the entire experimental period, which included HH diet and retro-orbital sinus injection containing 1 × 10^5^ MVs or MVEs, compared to C group (Table 2).

In terms of total cholesterol and triglyceride plasma levels, these were significantly increased for the HH group compared to hamsters from the C group, by 2.76 (*p* < 0.001) and 1.29 (*p* < 0.005) times, which means that the atherogenic diet induced hypercholesterolemia (Table 2).

For the hamsters from the HH-MVs or HH-MVEs groups, the total cholesterol and triglyceride plasma concentrations had significantly lower values than those of the HH group, by 1.48 (*p* < 0.001) and 2.53 times (*p* < 0.001), respectively, for the HH-MVs group, and by 1.57 (*p* < 0.001) and 1.82 times (*p* < 0.001), respectively, for the HH-MVEs group, which means that retro-orbital sinus injection containing 1 × 10^5^ MVs or MVEs attenuates diet-induced hypercholesterolemia (Table 2).

No significant change in body weight was observed for the diet-subjected animals and MVs/MVEs-injected animals when compared to those in the control group (Table 2).

To diagnose hypertension for our HH model, a quantitative analysis was performed based on individual hamster data, and it was found that systolic and diastolic blood pressures and heart rate were elevated for the HH group compared to hamsters from the C group, by 1.62 (*p* < 0.01), 1.64 (*p* < 0.001), and 1.57 (*p* < 0.05) times, respectively (Table 3). Injection with 1 × 10^5^ MVs or MVEs significantly reduced diet-induce hypertension in term of systolic and diastolic blood pressures and heart rate for hamsters in the HH-MVs group compared to the HH group by 1.19 (*p* < 0.005), 1.30 (*p* < 0.001), and 1.35 (*p* < 0.001) times, respectively, and in the HH-MVEs group compared to the HH group by 1.48 (*p* < 0.001), 1.49 (*p* < 0.001), and 1.40 (*p* < 0.001) times, respectively (Table 3).

To see functional modifications in the thoracic aorta, carotid artery, and left ventricle induced by the hyperlipemic-hypertensive diet or MV/MVE injections in HH hamsters, the hemodynamic parameters (distensibility, pulse wave velocity, wall thickness for arteries, and shortening fraction and relative wall thickness for left ventricle) were evaluated by echocardiography in the HH, HH-MVs, HH-MVEs groups compared with the C group. After analysis and quantification, the results revealed that all these investigated parameters were significantly modified for the HH group compared to the bC group (*p* < 0.001), while for the HH-MVs and HH-MVEs groups, these were close to the values from the C group, demonstrating the effectiveness of MV/MVE injection (Table 4). All these altered hemodynamic echocardiographic parameters are indicators of arterial dysfunction and stenosis, and also of ventricular hypertrophy in the HH group.

### 3.3. Beneficial Effects of MV and MVE Transplantation on the Circulating EPC, Cytokine, and Chemokine Levels

**Figure 4:**
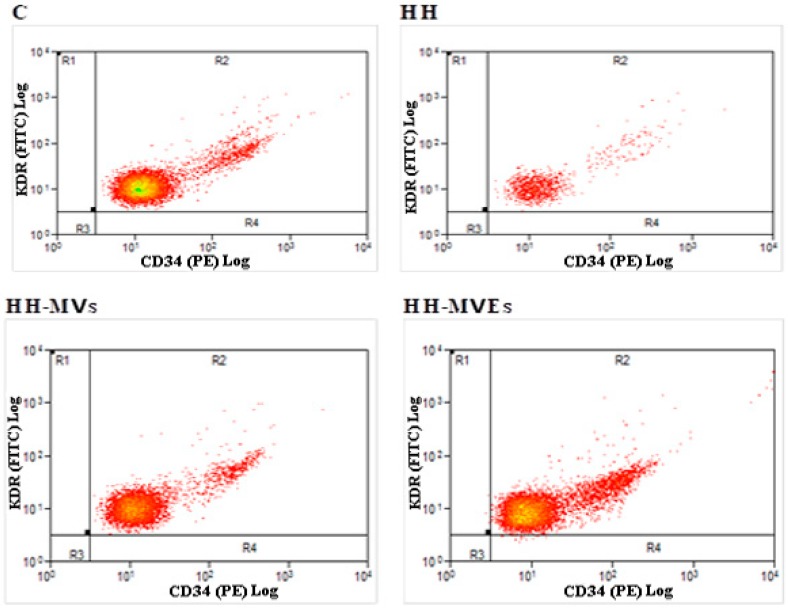
Specific identification of circulating endothelial progenitor cells (EPCs) isolated from all experimental groups, after 4 months of diet and treatment, by flow cytometry analysis; representative recordings for EPCs labeled with CD34 or KDR in the dark for 40 min at room temperature.

The specific identification and quantification of EPCs, expressed as percent of controls (100%), showed that the relative amounts of cells double positive for VEGF-R2^+^ (or KDR) and CD34^+^ were as follows: 100% for C, 10.75% ± 1.88% for HH, 56.55% ± 5.77% for HH-MVs, and 87.79% ± 9.03% for HH-MVEs (Table 5, Figure 4). The comparison of EPC (VEGF-R2^+^ and CD34^+^) levels revealed that the hyperlipemic–hypertensive diet in the HH group significantly diminished them compared with those from the C, HH-MVs, HH-MVEs groups (*p* < 0.001) (Table 5, Figure 4). On the other hand, simultaneous administration of MVs or MVEs with the diet mitigated the dramatic effects induced by the diet, keeping the levels of circulating EPCs at values close to normal, and in this way could promote their incorporation into the neoendothelium, an effect that is therapeutically implicated in diminishing neointimal formation (*p* < 0.001) (Table 5, Figure 4).

Because the plasma cytokine and chemokine levels are positively associated with atherosclerosis risk, these were measured by the ELISA method in the plasma collected from all experimental animals.

Our results showed that the plasma VEGF, MCP-1, IL-6, IL-1beta, IL-8, and CD-40L concentrations were significantly increased by the hyperlipemic–hypertensive diet in the HH group compared with those from the C group by 1.82 (*p* < 0.001), 1.68 (*p* < 0.005), 1.69 (*p* < 0.005), 1.8 (*p* < 0.001), 1.15 (*p* < 0.05), and 1.07 (*p* < 0.05) times respectively (Table 6). The administration of MVs or MVEs together with diet for 4 months (**1**) moderated the diet-induced increases of plasma VEGF, MCP-1, and IL-8 concentrations compared to the C group by 1.69 (*p* < 0.01), 1.61 (*p* < 0.01), and 1.03 (*p* < 0.05) times, respectively, for MVs and 1.52 (*p* < 0.01), 1.04 (*p* < 0.05), and 1.04 (*p* < 0.05) times, respectively, for MVEs and (**2**) prevented diet-induced increases of plasma IL-6 concentrations, the values being close to those measured for the C group (Table 6).

It is important to remark that MVE injection was much more efficient than MV injection; it had a greater contribution than MV injection to decreasing the circulating levels of VEGF, MCP-1, and IL-6 compared to the HH group (Table 6).

Interestingly, the circulating levels of IL-1beta and CD40L were increased in the HH hamsters transplanted with MVs or MVEs compared to the C group and even to the HH group (Table 6).

### 3.4. Role of MVs and MVEs on the Reversal of Structural and Functional Changes of Arterial Wall

To determine whether the hyperlipemic-hypertensive diet induces pathological vascular wall changes and, in contrast, whether MV or MVE administration reverses them, we performed histopathology and myography techniques in the thoracic aorta, carotid artery, and resistance artery isolated from hamsters in the C, HH, HH-MVs, and HH-MVEs experimental groups.

**Figure 5:**
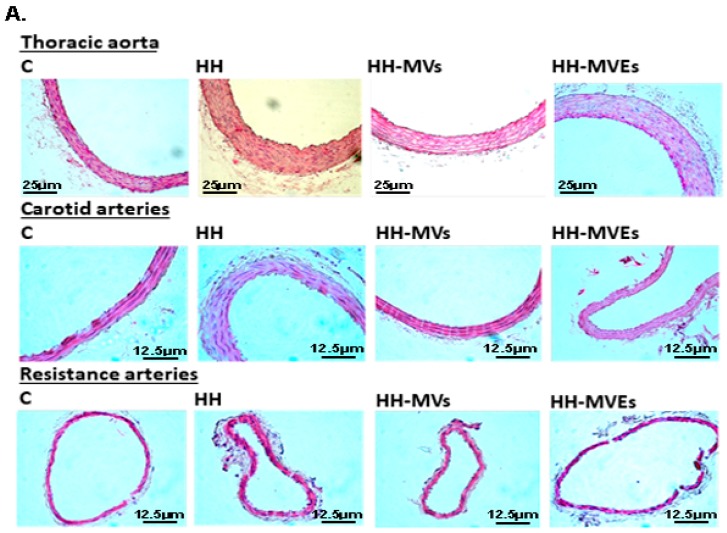

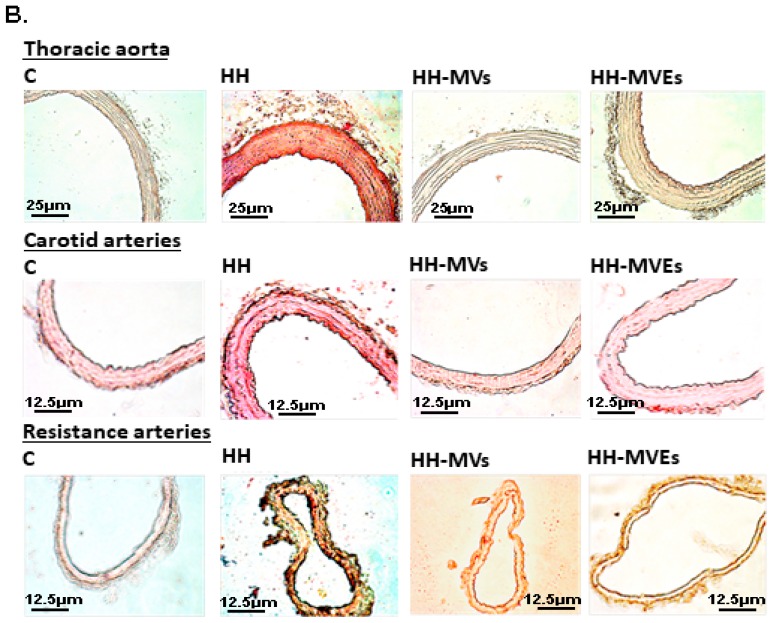
Structural changes of the vascular wall in the thoracic aorta, carotid artery, and resistance artery isolated from the C, HH, HH-MVs, and HH-MVEs groups (*n* = 5 individual animals per group): (**A**). The evaluation of morphology of the arterial wall by hematoxylin-eosin staining; (**B**). The representative micrographs showing oil-red O-stained lesions.

In HH hamsters fed with a hyperlipemic-hypertensive diet, we observed wall thickening and lesions displaying the morphological features of early stage lesions (Figure 5A). In addition, the lipid accumulation in the arterial wall from HH hamsters was detected according to oil red O staining (Figure 5B). Histological analysis of arterial walls from the HH-MVs and HH-MVEs groups did not show structural changes (Figure 5A,B), meaning that MV or MVE injection counterbalanced the diet effects.

In the present study, the role of hyperlipemic-hypertensive conditions on the vascular tone, such as contraction and relaxation of the vascular wall, was assessed through the myograph technique.

**Figure 6:**
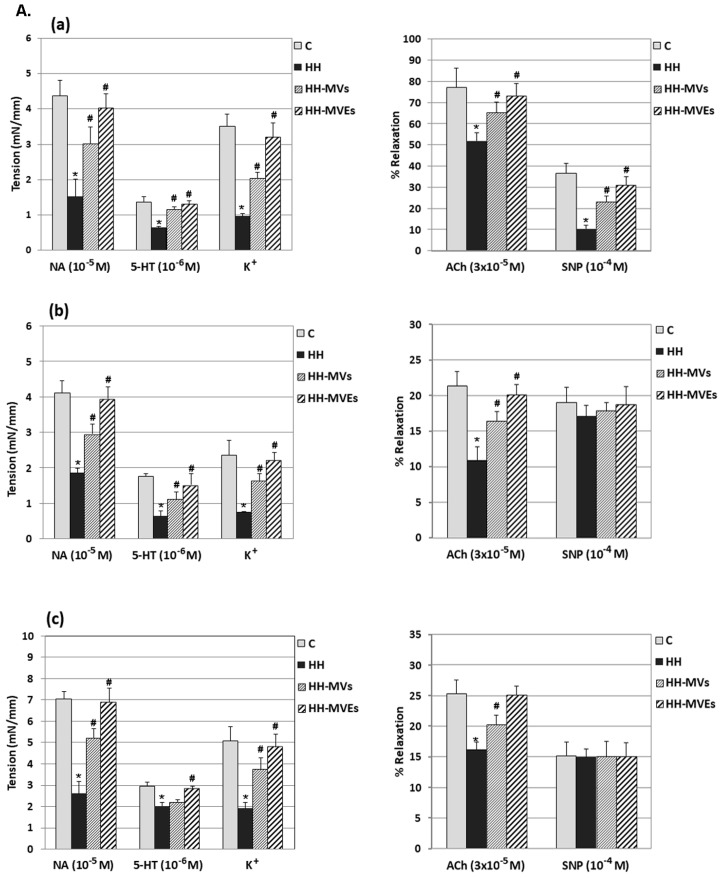

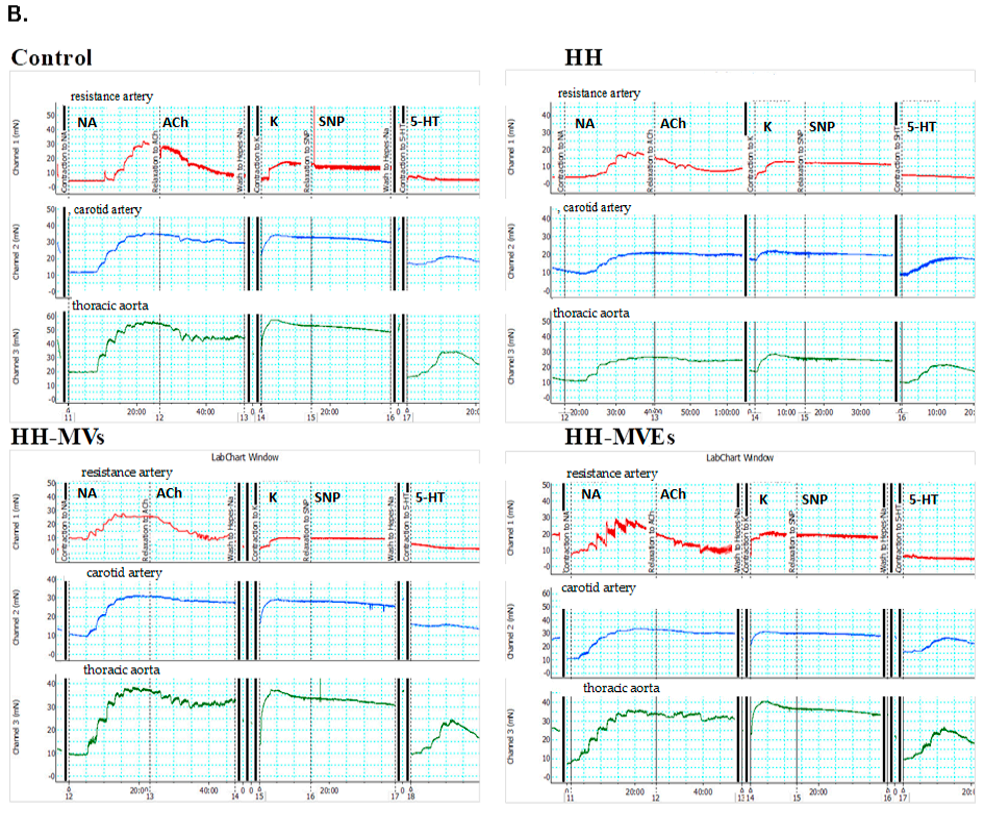
Functional investigation of the vascular wall of the thoracic aortas, carotid arteries and resistance arteries isolated from C, HH, HH-MVs, and HH-MVEs hamsters by the myograph technique (*n* = 8 individual animals per group): (**A**). The average maximal responses of contraction (to NA, 5-HT, K^+^) and relaxation (to ACh, SNP) of resistance arteries (a) carotid arteries (b), and thoracic aortas (c) explanted from C, HH, HH-MVs, HH-MVEs hamsters; (**B**). Representative recordings at selected time points for contraction (to NA, 5-HT, K^+^) and relaxation (to ACh, SNP) of resistance arteries, carotid arteries, and thoracic aortas from hamsters in the C, HH, HH-MVs, and HH-MVEs groups. The statistically significant differences between the groups were calculated and are represented as * *p* ≤ 0.05 for values vs. the C group and as # *p* ≤ 0.05 for values vs. the HH group (one way ANOVA analysis).

In the thoracic aortas, carotid arteries, and resistance arteries isolated from HH hamsters fed with a hyperlipemic-hypertensive diet, we found a significantly decreased contractile response to NA (10^−5^ M), 5-HT(10^−6^) and K^+^(83.93 mM) (*p* < 0.001) and also a significantly decreased endothelium dependent vasodilator response to ACh (3 × 10^−5^ M) (*p* < 0.001) compared to those measured at arteries from control hamsters (Figure 6A,B). As for endothelium independent vasodilator responses to SNP (10^−4^ M), these were significantly reduced for the resistance arteries in the HH group (*p* < 0.001) and unchanged for the carotid arteries and thoracic aortas in the same experimental group when the values were compared to those from control group (Figure 6A,B).

We also analyzed the effect of MV or MVE injection on the vascular tone of arteries isolated from the HH-MVs and HH-MVEs hamster groups. Isometric force measurements using the wire myograph technique revealed that the maximal contractile force developed by the thoracic aortas, carotid arteries and resistance arteries to NA (10^−5^ M), 5-HT (10^−6^), and K^+^(83.93 mM) were significantly increased compared with those obtained for arteries in the HH group (*p* < 0.005) and were similar to those recorded in the C group (Figure 6A,B). Arteries collected from hamsters in the HH-MVs and HH-MVEs groups developed a normal relaxation to ACh (3 × 10^−5^ M) and SNP (10^−4^ M), the values being similar to those in the C group (Figure 6A,B). These results demonstrate that MV/MVE administration may reduce the development of vascular dysfunction due to the hyperlipemic–hypertensive diet.

The beneficial effects of MV or MVE injection on the reversal of structural and functional vascular changes correlate well with those on hemodynamic and plasma parameters.

### 3.5. Validation of MVs and MVEs as Intercellular Carriers of miRNAs

First of all, the RNA integrity isolated from MVs or MVEs sorted from the peripheral blood collected from healthy hamsters was evaluated by electrophoresis.

**Figure 7:**
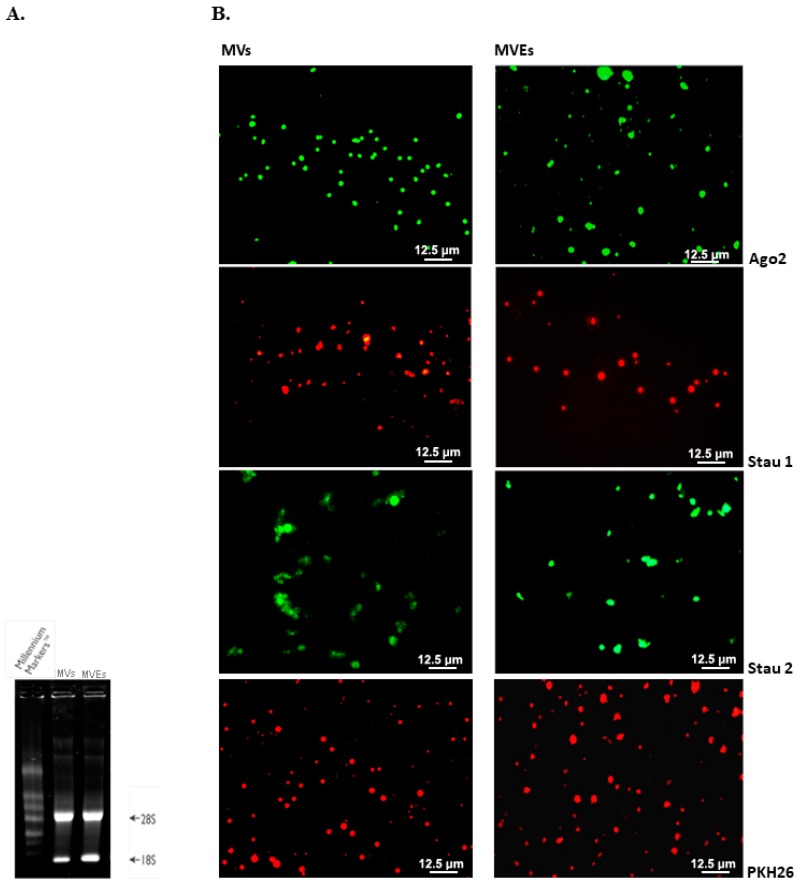
RNA and miRNA-binding proteins analysis in MVs or MVEs from healthy peripheral blood (*n* = 3 individual animals per group): (**A**). The presence of ribosomal subunits (rRNAs), 28S (5300 bp) and 18S (2000 bp), on 1% agarose gel demonstrates the integrity of isolated RNAs; (**B**). The appearance of fluorescence suggests that MVs/MVEs shuttle ribonucleoproteins (Ago2, Stau 1, Stau 2) bound to miRNA and involved in its traffic. In addition, the presence of PKH26 labeled MVs or MVEs on slides represents a positive control for the above samples.

For this, the presence of ribosomal subunits (rRNAs), 28S (5300 bp) and 18S (2000 bp), was examined on 1% denaturing agarose gel containing RNAs isolated from MVs or MVEs (Figure 7A). MVs/MVEs were found to contain various classes of RNA, with the major class represented by fragmented ribosomal RNA (rRNA), in particular 28S and 18S rRNA subunits (Figure 7A). In other words, RNAs have not been degraded and can be used in subsequent miRNA identification experiments in MVs or MVEs.

To prove that MVs or MVEs contain miRNAs, miRNA-binding proteins (Ago2, Stau1, and Stau2) were identified and quantified by indirect immunofluorescence (Figure 7B).

The presence of fluorescence on the slides containing 5 × 10^5^ MVs or MVEs sorted from the peripheral blood of healthy hamsters suggested that MVs and MVEs shuttle ribonucleoproteins involved in miRNA traffic such as Ago2 (a protein involved in miRNA transport and processing) and Stau 1 and 2 (staufen1/2, proteins involved in the transport and stability of mRNAs) (Figure 7B). A control showing the attachment of MVs or MVEs labeled with PKH26 (2 × 10^−6^ M) to the poly-*L*-lysine coated slides was performed and viewed under fluorescent microscope (Figure 7B).

**Figure 8:**
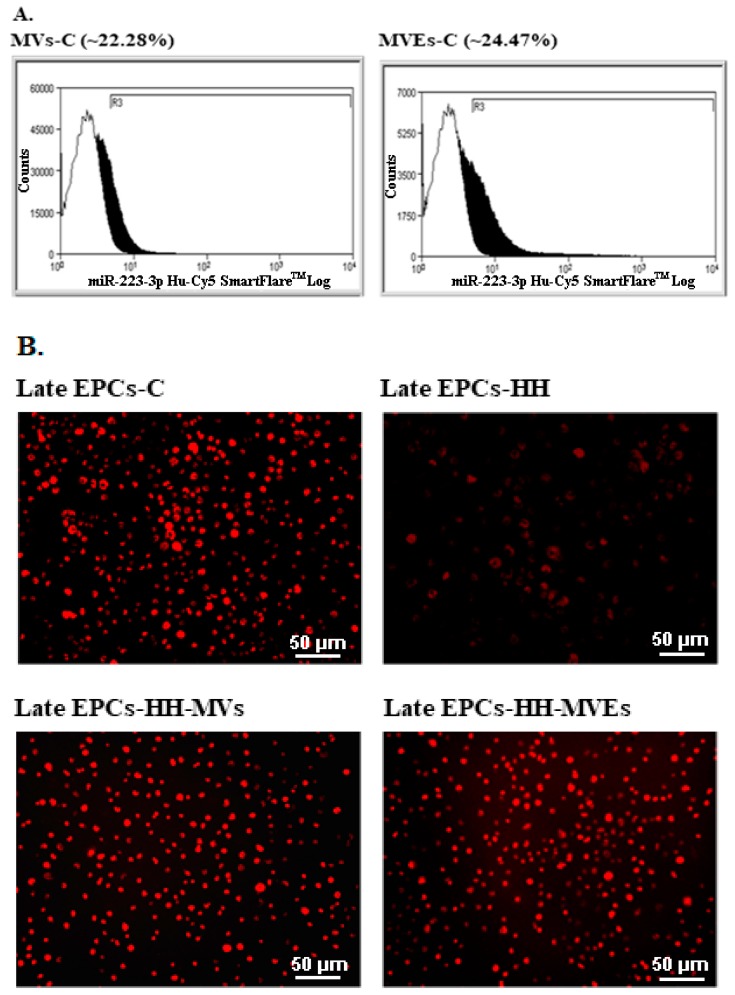
Evaluation of miR-223 expression by fluorescence detection using a miR-223-3p Hu-Cy5 SmartFlare (*n* = 5 individual animals per group): (**A**). The analysis of miR-223 expression in circulating MVs and MVEs by flow cytometry detection; (**B**). The assessment of miR-223 expression in late EPC cultures from the C, H, HH-MVs, and HH-MVEs animal groups by fluorescence microscopy.

The existence of miR-223, an important biomarker for metabolic diseases, was evaluated in 1 × 10^5^ MVs or MVEs obtained from blood collected from the C group using a miR-223-3p Hu-Cy5 SmartFlare™ miRNA detection probe. The presence of mir-223 in MVs and MVEs was demonstrated by flow cytometry analysis (Figure 8A).

In an attempt to find an explanation for the positive effects of MV or MVE administration on plasma, structural, and functional parameter changes in our atherosclerotic model (HH hamster), the miRNA transport from MVs or MVEs toward EPCs was evoked.

For this purpose, the late EPC cultures were obtained from the peripheral blood of C, HH, HH-MVs, HH-MVEs hamsters, and the miR-223 expression was assessed after use of the mir-223-3p Hu-Cy5 SmartFlare™ miRNA detection probe and fluorescence microscopy. As a consequence, the late EPCs-HH demonstrated significantly reduced miR-223 expression compared with late EPCs-C (*p* < 0.001) (Figure 8B). However, when taking MVs or MVEs treatment into consideration, we found that miR-223 expression at late EPCs-HH-MVs or late EPCs-HH-MVEs did not differ from late EPCs-C, meaning that MVs or MVEs transfer their miR-223 content towards circulating EPCs (Figure 8B).

### 3.6. Ability of MVs and MVEs from Control Group to Transfer miRNAs to Atherosclerotic Circulating EPCs

We continued to explore the mechanism by which MV or MVE treatment opposes diet-induced changes in the hypertensive-hyperlipidemic hamster model that mimics human atherosclerosis. As a result, the RNAs were extracted from 1 × 10^6^ circulating MVs or MVEs collected from the C and HH groups and 1 × 10^6^ circulating EPCs collected from the C, HH, HH-MVs, and HH-MVEs groups, and miRNAs such as miR-21, miR-126, miR-146a, and miR-223 with detrimental roles in cardiovascular disease were measured by RT q-PCR.

**Figure 9:**
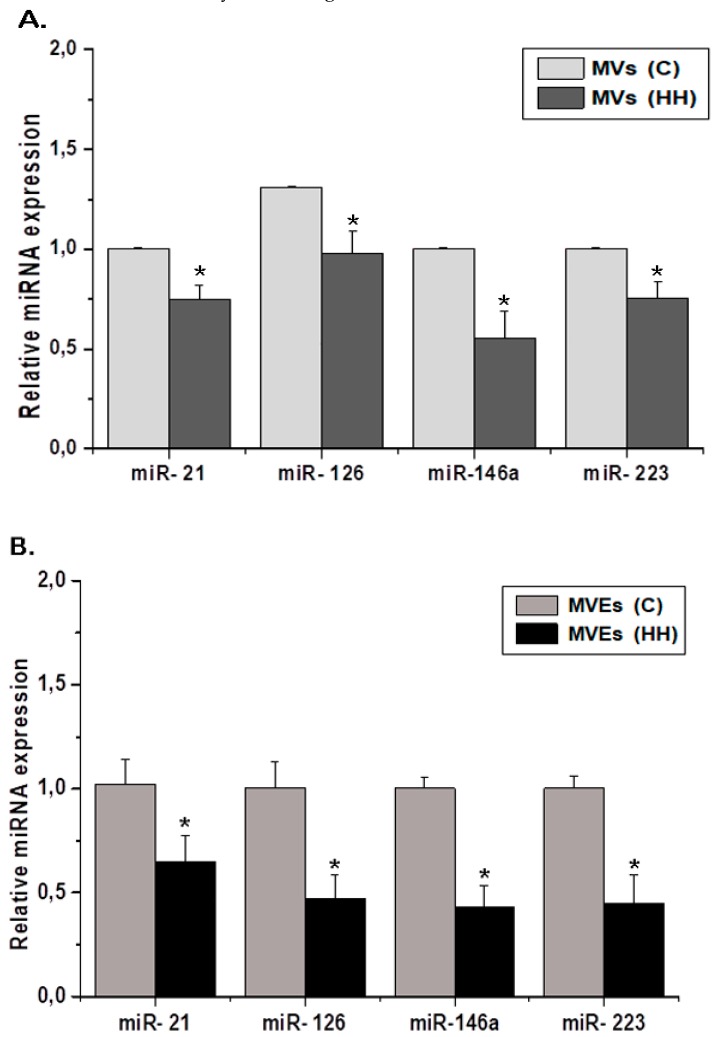

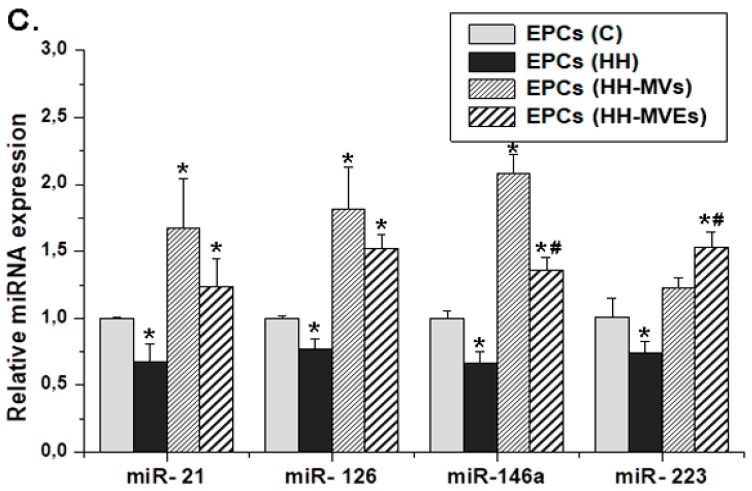
Evaluation of miRNA expressions in MVs (**A**) or MVEs (**B**) from the C and HH groups, and late EPCs from the C, H, HH-MVs, and HH-MVEs animal groups (**C**) by qRT-PCR (*n* = 6 individual animals per group); the relative quantification of miR-21, miR-126, miR-146a, and miR-223. The statistically significant differences between the groups were calculated and are represented as * *p* ≤ 0.05 for values vs. the C group and as # *p* ≤ 0.05 for values vs. the HH group (one way ANOVA analysis).

The results showed that the miRNA levels were significantly lower in MVs or MVEs from the HH group compared to MVs and MVEs from the C group, and also in late EPCs from the HH group compared to late EPCs from the C group (*p* ≤ 0.05) (Figure 9A,B). In addition, the results demonstrated that MV or MVE administration of healthy origin increases significantly the miR-21, miR-126, miR-146a, and miR-223 levels in late EPCs from the HH-MVs and HH-MVEs groups compared to late EPCs from the HH group, where the levels of these miRNAs were significantly reduced compared to those from late EPCs in the C group (*p* ≤ 0.01) (Figure 9C). These data indicate that healthy MVs and MVEs are capable of transferring miRNA into dysfunctional hypertensive-hyperlipidemic EPCs after their intravenous injection, improving in this way some cardiometabolic disorders induced by an atherogenic diet.

## 4. Discussion

Cardiovascular diseases (CVD) are the principal cause of mortality worldwide. There are a number of known risk factors that trigger the occurrence of hypertension, atherosclerosis, and, implicitly, of CVD. While cardiovascular risk factors are what initiate the disease, biomarkers that have an important biological basis contribute to the cardiovascular risk and disease course. As a result, a correct diagnosis for patients with CVD could be established by taking into account both risk factors and disease specific biomarkers.

The discovery of MVs in biological fluids, particularly in peripheral blood, has opened a new era and new perspectives in diagnosis, prognosis, and therapy of CVD pathogenesis. It is known that MVs have a dual behavior, namely, that they can be important biomarkers for CVD and, as a result, a real target for therapy, but at the same time they can also be used as therapeutic agents in their natural form or can be genetically manipulated [9].

Given the natural property of MVs for genetic information transfer, we thought to investigate the possibility of exploiting MVs for therapeutic purposes in atherosclerosis as a major cause of CVD. To this end, we conducted a study designed to assess the impact of therapy with MVs, in particular with EPCs-derived MVs (MVEs), of healthy origins on pathological changes in plasma parameters and the vascular wall induced by a hypertensive–hyperlipidemic diet in an atherosclerotic animal model (HH hamsters), and to clarify the involved mechanisms. Thus, on our experimental model of atherosclerotic vascular disease, MVs and EPC-derived MVs have a central role as transfer vehicles of miRNAs in circulation, specific of the origin cell and disease.

Our data showed the following main findings: MVs and MVEs of healthy origins transplanted to HH hamsters counteract the hypertensive-hyperlipidemic diet-induced detrimental effects on plasma, structural, and functional parameters by biologically active miR-10a, miR-21, miR-126, and miR-146a transfer to circulating EPCs, mediating their vascular repair function in the atherosclerosis processes.

There is growing evidence that MVs are not inert particles, they are now associated with a number of important physiological and pathological processes, such as inflammatory processes connected with CVD or not, due to their biological content that they secrete [34,35,36].

A recent study showed that MVs are also associated with key steps in atherosclerosis, including cellular lipid metabolism, endothelial dysfunction, and vascular wall inflammation, ultimately resulting in vascular remodeling [37]. In close connection with this study, it was shown that circulating MVs, in particular platelet MVs and endothelial MVs, were significantly increased in a variety of thrombotic disorders, being positively associated with disease pathophysiology, its activity, or progression [6,38]. The endothelial MV’s plasma levels have been found to be in close contact with risk factors and CVD. Circulating platelet MVs were also shown to be involved in the progressive formation of atherosclerotic plaque and arterial thrombosis development [39,40], especially in diabetic patients [41]. MVs are now seen as early, non-invasive biomarkers for numerous disorders and because of their content in miRNAs, they can directly contribute to the development of vascular complications in atherosclerosis and diabetes [19].

In our previous studies on atherosclerotic animal models and in patients with hypertension and dyslipidemia, we showed that treatment with irbesartan, an AT1 receptor antagonist, diminished total MV levels in peripheral blood, especially specific MVs (leukocyte-, platelet-, and endothelial-derived MVs), and increased EPC levels, thereby preventing the development of vascular endothelial dysfunction by the augmentation of endothelium-mediated vasodilation [38,42,43,44]. In addition, the irbersartan treatment modified the protein expressions of specific membrane receptors exposed by MVs (TF, P-Selectin, E-Selectin, PSGL-1, Rantes) and EPCs (β2-Integrins, α4β1-integrin) and of the VEGF/SDF-1α axis [30].

In all of these studies, MVs were treated as biomarkers and as a result, real targets for drugs, but studies regarding the use of MVs as therapeutic vectors in some pathologies, including CVD, are few and have only been completed on animal models. Concerning the use of MVEs as therapeutic tools, as far as we know, there are very few studies in this regard.

Thus, one of the aims of our work was to understand deeply the mysteries of CVD, particularly MV and miRNA involvement, and to provide proof-of-concept that MVs, particularly EPC-derived MVs, by their ability to transport a subset of miRNAs into circulation and transfer them to target cells (EPCs, ECs), might represent a valuable new therapeutic strategy for regenerative medicine in CVD. In addition, MVs released from EPCs could mimic the effect of origin cells, suggesting that they could be a more effective therapeutic approach. Also, our study opens up the possibility of transferring miRNAs or small interfering RNAs (siRNAs) via MVs/MVEs in different pathologies.

There are studies that have shown that that abnormal miRNA expression in MVs leads to neoangiogenesis. These were mentioned in our recently published chapter [45]. Thus, it has been demonstrated that reduced expression of miRNA-200b decreased VEGF levels [46], and increased expression of miR-29b augmented VEGF levels [47]. These data show that two different miRs of MVs have opposite effects on the same molecule, VEGF, with an extremely important role in angiogenesis. Moreover, the data suggested that acting on these miRNA levels in MVs may regulate cell proliferation in diabetic retinopathy. Likewise, other studies showed that administration of the miR-126-enriched MVs to ApoE^−^/^−^ mice diminished aortic plaque progression in atherosclerosis [48], and miR-126 in MVs has a substantial involvement in angiogenesis and vascular integrity [49]. Notably, it has been shown that MVs released from EPCs, transporting specific mRNAs, stimulate angiogenesis via the phosphatidylinositol 3 kinase/protein kinase B signaling pathway [49]. In line with these results, in our recent published paper we found that MVs of healthy origins advance in vitro EPC proliferation, adhesion, and migration by transfer of miR21, miR-126, miR-10a, miR-146a, and miR-223 into recipient cells and by insulin-like growth factor-1 expression activation [33]. These data extrapolated to the in vivo system raised the question if MVs of healthy origins by the transfer of miRNAs could increase EPC’s ability to adhere to the endothelial lesion area, transform into adult endothelial cells, and restore vascular endothelium under atherosclerotic conditions. For this reason, in order to answer this question, we initiated the present in vivo study.

The realization of this study was also based on our recent experimental results showing that platelets of healthy origins support functional improvement of atherosclerotic EPCs [50].

Consequently, in addition to our previous in vitro studies, the present preclinical in vivo study indicates the following: (**A**) MV and MVE transplantation of healthy origins significantly reduces atherosclerosis process development via (**1**) the mitigation of dyslipidemia, hypertension, circulating EPC levels, and cytokine/chemokine profiles (VEGF, IL-6, IL-8) and (**2**) the structural and functional remodeling within the vessel wall and heart in terms of distensibility/stiffness/pulse wave velocity of the thoracic aorta, carotid wall thickness, systolic and diastolic function of the left ventricle, left ventricular hypertrophy, lipid accumulation/contraction/relaxation in the thoracic aorta, carotid, and resistance arteries; (**B**) circulating MVs contain miRNAs, this was demonstrated by validating MVs and MVEs as transporters of Ago2-miRNA, Stau1-miRNA, and Stau2-miRNA complexes, and (**C**) MV and MVE administrations significantly protect against atherosclerotic vascular disease via transfer of miR-223, miR-21, miR-126, and miR-146a to circulating late EPCs. Moreover, with this study we showed that MVs, mainly MVEs, reproduced the favorable role of their parent cells of healthy origins in the treatment of atherosclerosis, possibly via microRNA transfer, and the beneficial effects of MVEs are greater than those of MVs.

Our findings introduce some new information about MVs and EPCs, thus contributing to a better understanding of MV/miRNA/EPC contribution to vascular dysfunction development/reversion and atherosclerotic plaque progression/regression. Specifically, our results show that intravenous administration of allogenic MVs and MVEs prevents/slows down atherosclerotic cardiovascular disease development in HH hamsters, impacting plasma parameters, left ventricle vasculature, and EPC mobilization. In addition, the results of our study support and supplement the information regarding MVs as protective and major transport vehicles for distinct miRNAs in circulation associated with CVD. We will also bring to light evidence concerning the differences in miRNA transfer by MVs and EPC-derived MVs.

According to the results of our study, we assume that MVs and EPC-derived MVs (MVEs) may be capable of manipulating stem/progenitor cells to repair tissue injury by miRNA transfer. This is a hypothesis that is very difficult to validate in vivo, and other mechanisms may be involved, for example, MVs or MVEs could carry molecules other than miRNAs that could increase the expression levels of endogenous miRNAs in the circulating EPCs. As a consequence, MVs and MVEs could induce epigenetic changes in EPCs by delivering their content (miRNAs or other molecules), reactivating or modulating their tissue regenerative programs.

Thus, these results suggest that MVs and especially MVEs could indeed be exploited as a new therapeutic approach in atherosclerotic CVD, thus giving hope to patients with CVD.

## Figures and Tables

**Table 1:** Quantification of radiant efficiency for PKH26 (Ex = 535 nm; Em = 580 nm) in liver, lung, kidney, brain, heart, thoracic aorta, and mesenteric resistance arteries by the measurement of fluorescence signals originating from MVs and MVEs labelled with PKH26 and transplanted to the HH group (transplanted HH group) under an IVIS imaging system.

Organs/Tissues from Transplanted HH Group	Liver	Heart	Lung	Kidney	Brain	Thoracic Aorta	Mesenteric Arteries
Radiant Efficiency = p/sec/cm^2^/sr µW/cm^2^ For MVs-PKH26	4.52 × 10^9^ ± 0.10	6.73 × 10^7^ ± 1.02	1.95 × 10^9^ ± 0.04	4.27 × 10^8^ ± 0.22	2.87 × 10^8^ ± 0.96	7.47 × 10^8^ ± 0.15	2.37 × 10^7^ ± 0.09
Radiant Efficiency = p/sec/cm^2^/sr µW/cm^2^ For MVEs-PKH26	4.79 × 10^9^ ± 0.17	6.89 × 10^7^ ± 1.45	2.01 × 10^9^ ± 0.11	4.34 × 10^8^ ± 0.36	2.91 × 10^8^ ± 0.98	7.53 × 10^8^ ± 0.23	2.52 × 10^7^ ± 0.13

**Table 2:** Levels of body weight (g) and plasma glucose, total cholesterol, and triglycerides, all expressed as mg/dl, in the HH, HH-MVs, and HH-MVEs experimental groups versus C group. The data correspond to the analysis done at 4 months of hyperlipemic–hypertensive diet and MV/MVE treatment.

Parameters	C (*n* = 8)	HH (*n* = 14)	HH-MVs (*n* = 6)	HH-MVEs (*n* = 6)
**Body Weight (g)**	115.2 ± 2.5	105.3 ± 3.4	114.7 ± 2.2	116.4 ± 3.1
**Glucose (mg/dl)**	96.75 ± 2.90	94.47 ± 2.30	86.16 ± 5.28	86.33 ± 6.21
**Total Cholesterol (mg/dl)**	146.04 ± 5.46	403.67 ± 16.46	271.81 ± 9.80	256.21 ± 7.788
**Triglyceride (mg/dl)**	153.27 ± 10.57	499.59 ± 45.16	197.14 ± 22.35	273.32 ± 26.61

**Table 3:** Blood pressure readings as indicators of health or hypertension for hamster experimental groups: C, HH, HH-MVs, and HH-MVEs. The data correspond to the analysis done after 4 months of diet and treatment.

Parameters	C (*n* = 5)	HH (*n* = 5)	HH-MVs (*n* = 5)	HH-MVEs (*n* = 5)
**Systolic Arterial Blood Pressure (mm HG)**	90.83 ± 2.42	147.45 ± 2.75	123.14 ± 2.55	99.25 ± 2.15
**Diastolic Arterial Blood Pressure (mm HG)**	67.82 ± 2.91	111.05 ± 3.71	85.37 ± 3.95	74.17 ± 3.76
**Heart Rate (BMP)**	312 ± 7	489 ± 19	361 ± 12	347 ± 11

**Table 4:** Structural/architectural and flow changes of thoracic aorta, carotid artery and left ventricle as a measure of vascular rigidity and ventricular hypertrophy: diastolic diameter (AoD) and systolic diameter (AoS) of the ascending aorta or carotid artery, pulse wave velocity (PWV) for thoracic aorta and carotid artery, shortening fraction (SF) of left ventricle, diastolic diameter of left ventricle LVID(d), systolic diameter of left ventricle LVID(s), relative wall thickness of left ventricle (RWT), posterior wall thickness of left ventricle (PWTd). The data correspond to the analysis done at 4 months of diet and treatment.

Measurements by Duplex Ultrasonography Using Vevo2100	C (*n* = 5)	HH (*n* = 5)	HH-MVs (*n* = 5)	HH-MVEs (*n* = 5)
**Thoracic Aortic Distensibility (AoS-AoD) (mm)**	0.66 ± 0.06	0.13 ± 0.03	0.43 ± 0.05	0.44 ± 0.04
**PWV for Thoracic Aorta (mm/sec)**	620.09 ± 23.45	2215.26 ± 121.72	742.85 ± 45.87	653.28 ± 42.91
**Carotid Wall Thickness (mm)**	0.037 ± 0.003	0.109 ± 0.007	0.068 ± 0.005	0.045 ± 0.005
**PWV for Carotid Arteries (mm/sec)**	841.012 ± 54.93	1476.49 ± 97.36	1102.026 ± 81.77	996.23 ± 72.17
**SF = Shortening Fraction = LVID(d)–LVID(s) = for Systolic Function of Left Ventricle (mm)**	1.58 ± 0.41	0.78 ± 0.13	2.37 ± 0.98	1.72 ± 0.55
**RWT = Relative Wall Thickness of Left Ventricle = (2xPWTd)/LVIDd**	0.44 ± 0.03	0.63 ± 0.05	0.38 ± 0.02	0.67 ± 0.06

**Table 5:** Quantification (in percentages of control) of circulating endothelial progenitor cells (EPCs) (CD34^+^, KDR^+^) by flow cytometry analysis, as a biomarker of cardiovascular disease after 4 months of the hyperlipemic–hypertensive diet and MV/MVE treatment.

EPCs	C (*n* = 8)	HH (*n* = 14)	HH-MVs (*n* = 6)	HH-MVEs (*n* = 6)
**CD34^+^, KDR^+^ (%)**	100	10.75 ± 1.88	56.55 ± 5.77	87.79% ± 9.03

**Table 6:** Analysis of plasma cytokine and chemokine profiles, inflammatory biomarkers with a role in the pathogenesis of vascular complications, by the ELISA method for all experimental groups (C, HH, HH-MVs, HH-MVEs).

Plasmatic Parameters	C (*n* = 8)	HH (*n* = 14)	HH-MVs (*n* = 6)	HH-MVEs (*n* = 6)
**VEGF (pg/mL)**	44.95 ± 3.08	81.81 ± 5.43	76.07 ± 5.89	68.37 ± 7.40
**MCP-1 (pg/mL)**	1052.13 ± 90.92	1770.58 ± 152.81	1700.91 ± 99.21	1101.92 ± 223.19
**IL-6 (pg/mL)**	5.50 ± 0.22	9.28 ± 0.60	4.36 ± 0.42	3.96 ± 0.24
**IL-1beta (pg/mL)**	4.41 ± 1.72	7.94 ± 2.22	20.42 ± 3.26	29.29 ± 6.34
**Il-8 (pg/mL)**	347.80 ± 8.63	399.29 ± 4.09	357.94 ± 16.62	363.79 ± 3.99
**CD40L (pg/mL)**	396.52 ± 11.31	425.88 ± 34.30	430.97 ± 30.78	467.96 ± 17.11

## References

[1] Libby P., Aikawa M. (2002). Stabilization of atherosclerotic plaques: New mechanisms and clinical targets. Nat. Med..

[2] Hansson G.K. (2005). Inflammation, atherosclerosis, and coronary artery disease. N. Engl. J. Med..

[3] Baron M., Boulanger C.M., Staels B., Tailleux A. (2012). Cell-derived microparticles in atherosclerosis: Biomarkers and targets for pharmacological modulation?. J. Cell. Mol. Med..

[4] Georgescu A., Alexandru N., Popov D., Amuzescu M., Andrei E., Zamfir C., Maniu H., Badila A. (2009). Chronic venous insufficiency is associated with elevated level of circulating microparticles. J. Thromb. Haemost. JTH.

[5] Shantsila E., Kamphuisen P.W., Lip G.Y. (2010). Circulating microparticles in cardiovascular disease: Implications for atherogenesis and atherothrombosis. J. Thromb. Haemost. JTH.

[6] Georgescu A., Alexandru N., Andrei E., Titorencu I., Dragan E., Tarziu C., Ghiorghe S., Badila E., Bartos D., Popov D. (2012). Circulating microparticles and endothelial progenitor cells in atherosclerosis: Pharmacological effects of irbesartan. J. Thromb. Haemost. JTH.

[7] Hugel B., Martinez M.C., Kunzelmann C., Freyssinet J.M. (2005). Membrane microparticles: Two sides of the coin. Physiology.

[8] Prokopi M., Pula G., Mayr U., Devue C., Gallagher J., Xiao Q., Boulanger C.M., Westwood N., Urbich C., Willeit J. (2009). Proteomic analysis reveals presence of platelet microparticles in endothelial progenitor cell cultures. Blood.

[9] Alexandru N., Costa A., Constantin A., Cochior D., Georgescu A. (2017). Microparticles: From Biogenesis to Biomarkers and Diagnostic Tools in Cardiovascular Disease. Curr. Stem Cell Res. Ther..

[10] Mause S.F., Weber C. (2010). Microparticles: Protagonists of a novel communication network for intercellular information exchange. Circ. Res..

[11] Cai X., Hagedorn C.H., Cullen B.R. (2004). Human microRNAs are processed from capped, polyadenylated transcripts that can also function as mRNAs. RNA.

[12] Ratajczak J., Miekus K., Kucia M., Zhang J., Reca R., Dvorak P., Ratajczak M.Z. (2006). Embryonic stem cell-derived microvesicles reprogram hematopoietic progenitors: Evidence for horizontal transfer of mRNA and protein delivery. Leukemia.

[13] Lewis B.P., Burge C.B., Bartel D.P. (2005). Conserved seed pairing, often flanked by adenosines, indicates that thousands of human genes are microRNA targets. Cell.

[14] Berezikov E., Guryev V., van de Belt J., Wienholds E., Plasterk R.H., Cuppen E. (2005). Phylogenetic shadowing and computational identification of human microRNA genes. Cell.

[15] Gupta S.K., Bang C., Thum T. (2010). Circulating microRNAs as biomarkers and potential paracrine mediators of cardiovascular disease. Circ. Cardiovasc. Genet..

[16] Bauersachs J., Thum T. (2011). Biogenesis and regulation of cardiovascular microRNAs. Circ. Res..

[17] Fichtlscherer S., Zeiher A.M., Dimmeler S. (2011). Circulating microRNAs: Biomarkers or mediators of cardiovascular diseases?. Arterioscler. Thromb. Vasc. Biol..

[18] Hartmann D., Thum T. (2011). MicroRNAs and vascular (dys)function. Vasc. Pharmacol..

[19] Alexandru N., Badila E., Weiss E., Cochior D., Stepien E., Georgescu A. (2016). Vascular complications in diabetes: Microparticles and microparticle associated microRNAs as active players. Biochem. Biophys. Res. Commun..

[20] Cipollone F., Felicioni L., Sarzani R., Ucchino S., Spigonardo F., Mandolini C., Malatesta S., Bucci M., Mammarella C., Santovito D. (2011). A unique microRNA signature associated with plaque instability in humans. Stroke.

[21] Raitoharju E., Lyytikainen L.P., Levula M., Oksala N., Mennander A., Tarkka M., Klopp N., Illig T., Kahonen M., Karhunen P.J. (2011). miR-21, miR-210, miR-34a, and miR-146a/b are up-regulated in human atherosclerotic plaques in the Tampere Vascular Study. Atherosclerosis.

[22] Bidzhekov K., Gan L., Denecke B., Rostalsky A., Hristov M., Koeppel T.A., Zernecke A., Weber C. (2012). microRNA expression signatures and parallels between monocyte subsets and atherosclerotic plaque in humans. Thromb. Haemost..

[23] Nemecz M., Alexandru N., Tanko G., Georgescu A. (2016). Role of MicroRNA in Endothelial Dysfunction and Hypertension. Curr. Hypertens. Rep..

[24] Shi M.A., Shi G.P. (2010). Intracellular delivery strategies for microRNAs and potential therapies for human cardiovascular diseases. Sci. Signal..

[25] Diehl P., Fricke A., Sander L., Stamm J., Bassler N., Htun N., Ziemann M., Helbing T., El-Osta A., Jowett J.B. (2012). Microparticles: Major transport vehicles for distinct microRNAs in circulation. Cardiovasc. Res..

[26] Lacroix R., Plawinski L., Robert S., Doeuvre L., Sabatier F., Martinez de Lizarrondo S., Mezzapesa A., Anfosso F., Leroyer A.S., Poullin P. (2012). Leukocyte- and endothelial-derived microparticles: A circulating source for fibrinolysis. Haematologica.

[27] Gu S., Zhang W., Chen J., Ma R., Xiao X., Ma X., Yao Z., Chen Y. (2014). EPC-derived microvesicles protect cardiomyocytes from Ang II-induced hypertrophy and apoptosis. PLoS ONE.

[28] Leroyer A.S., Ebrahimian T.G., Cochain C., Recalde A., Blanc-Brude O., Mees B., Vilar J., Tedgui A., Levy B.I., Chimini G. (2009). Microparticles from ischemic muscle promotes postnatal vasculogenesis. Circulation.

[29] Heiss C., Keymel S., Niesler U., Ziemann J., Kelm M., Kalka C. (2005). Impaired progenitor cell activity in age-related endothelial dysfunction. J. Am. Coll. Cardiol..

[30] Georgescu A., Alexandru N., Nemecz M., Titorencu I., Popov D. (2013). Irbesartan administration therapeutically influences circulating endothelial progenitor cell and microparticle mobilization by involvement of pro-inflammatory cytokines. Eur. J. Pharmacol..

[31] Mulvany M.J., Halpern W. (1977). Contractile properties of small arterial resistance vessels in spontaneously hypertensive and normotensive rats. Circ. Res..

[32] Frohlich E., Meindl C., Roblegg E., Ebner B., Absenger M., Pieber T.R. (2012). Action of polystyrene nanoparticles of different sizes on lysosomal function and integrity. Part. Fibre Toxicol..

[33] Alexandru N., Andrei E., Niculescu L., Dragan E., Ristoiu V., Georgescu A. (2017). Microparticles of healthy origins improve endothelial progenitor cell dysfunction via microRNA transfer in an atherosclerotic hamster model. Acta Physiol..

[34] Lawson C., Vicencio J.M., Yellon D.M., Davidson S.M. (2016). Microvesicles and exosomes: New players in metabolic and cardiovascular disease. J. Endocrinol..

[35] Baroni S., Romero-Cordoba S., Plantamura I., Dugo M., D’Ippolito E., Cataldo A., Cosentino G., Angeloni V., Rossini A., Daidone M.G. (2016). Exosome-mediated delivery of miR-9 induces cancer-associated fibroblast-like properties in human breast fibroblasts. Cell Death Dis..

[36] Blin J., Fitzgerald K.A. (2015). Perspective: The RNA exosome, cytokine gene regulation and links to autoimmunity. Cytokine.

[37] Deng W., Tang T., Hou Y., Zeng Q., Wang Y., Fan W., Qu S. (2019). Extracellular vesicles in atherosclerosis. Clin. Chim. Acta Int. J. Clin. Chem..

[38] Orbe J., Alexandru N., Roncal C., Belzunce M., Bibiot P., Rodriguez J.A., Meijers J.C., Georgescu A., Paramo J.A. (2015). Lack of TAFI increases brain damage and microparticle generation after thrombolytic therapy in ischemic stroke. Thromb. Res..

[39] Alexandru N., Popov D., Dragan E., Andrei E., Georgescu A. (2013). Circulating endothelial progenitor cell and platelet microparticle impact on platelet activation in hypertension associated with hypercholesterolemia. PLoS ONE.

[40] Georgescu A., Alexandru N., Andrei E., Dragan E., Cochior D., Dias S. (2016). Effects of transplanted circulating endothelial progenitor cells and platelet microparticles in atherosclerosis development. Biol. Cell.

[41] Stepien E.L., Durak-Kozica M., Kaminska A., Targosz-Korecka M., Libera M., Tylko G., Opalinska A., Kapusta M., Solnica B., Georgescu A. (2018). Circulating ectosomes: Determination of angiogenic microRNAs in type 2 diabetes. Theranostics.

[42] Falk R.H., Camm A.J. (2009). Rethinking the reasons to treat atrial fibrillation? The role of dronedarone in reducing cardiovascular hospitalizations. Eur. Heart J..

[43] Tokarz A., Szuscik I., Kusnierz-Cabala B., Kapusta M., Konkolewska M., Zurakowski A., Georgescu A., Stepien E. (2015). Extracellular vesicles participate in the transport of cytokines and angiogenic factors in diabetic patients with ocular complications. Folia Med. Crac..

[44] Bădila E., Daraban A.M., Ghiorghe S., Georgescu A., Alexandru N., Bartoş D., Tîrziu C. (2014). Rethinking cardiovascular therapy—The effect of irbesartan on circulating microparticles and endothelial progenitor cells in patients with hypertension and dyslipidemia. Farmacia.

[45] Gherghiceanu M., Alexandru N., Magda S.L., Constantin A., Nemecz M., Filippi A., Ioghen O.C., Ceafalan L.C., Bojin F., Tanko G., de Bona A.G. (2019). Extracellular vesicles as valuable players in diabetic cardiovascular diseases. Extracellular Vesicles.

[46] McArthur K., Feng B., Wu Y., Chen S., Chakrabarti S. (2011). MicroRNA-200b regulates vascular endothelial growth factor-mediated alterations in diabetic retinopathy. Diabetes.

[47] Silva V.A., Polesskaya A., Sousa T.A., Correa V.M., Andre N.D., Reis R.I., Kettelhut I.C., Harel-Bellan A., De Lucca F.L. (2011). Expression and cellular localization of microRNA-29b and RAX, an activator of the RNA-dependent protein kinase (PKR), in the retina of streptozotocin-induced diabetic rats. Mol. Vis..

[48] Zernecke A., Bidzhekov K., Noels H., Shagdarsuren E., Gan L., Denecke B., Hristov M., Koppel T., Jahantigh M.N., Lutgens E. (2009). Delivery of microRNA-126 by apoptotic bodies induces CXCL12-dependent vascular protection. Sci. Signal..

[49] Chen Y., Li G., Liu M.L. (2018). Microvesicles as Emerging Biomarkers and Therapeutic Targets in Cardiometabolic Diseases. Genom. Proteom. Bioinform..

[50] Alexandru N., Safciuc F., Constantin A., Nemecz M., Tanko G., Filippi A., Dragan E., Badila E., Georgescu A. (2019). Platelets of Healthy Origins Promote Functional Improvement of Atherosclerotic Endothelial Progenitor Cells. Front. Pharmacol..

